# Potency of Fusion-Inhibitory Lipopeptides against SARS-CoV-2 Variants of Concern

**DOI:** 10.1128/mbio.01249-22

**Published:** 2022-06-13

**Authors:** Katharina S. Schmitz, Daryl Geers, Rory D. de Vries, T. Francesca Bovier, Anna Z. Mykytyn, Corine H. Geurts van Kessel, Bart L. Haagmans, Matteo Porotto, Rik L. de Swart, Anne Moscona

**Affiliations:** a Department Viroscience, Erasmus MC, Rotterdam, the Netherlands; b Department of Pediatrics, Columbia University Vagelos College of Physicians & Surgeons, New York, New York, USA; c Center for Host–Pathogen Interaction, Columbia University Vagelos College of Physicians & Surgeons, New York, New York, USA; d Department of Experimental Medicine, University of Campania Luigi Vanvitelli, Caserta, Italy; e Department of Physiology & Cellular Biophysics, Columbia University Vagelos College of Physicians & Surgeons, New York, New York, USA; f Department of Microbiology & Immunology, Columbia University Vagelos College of Physicians & Surgeons, New York, New York, USA; St. Jude Children's Research Hospital

**Keywords:** SARS-CoV-2, Spike protein, fusion inhibitor, postvaccine sera, viral evolution

## Abstract

The ability of SARS-CoV-2 to evolve in response to selective pressures poses a challenge to vaccine and antiviral efficacy. The S1 subunit of the spike (S) protein contains the receptor-binding domain and is therefore under selective pressure to evade neutralizing antibodies elicited by vaccination or infection. In contrast, the S2 subunit of S is only transiently exposed after receptor binding, which makes it a less efficient target for antibodies. As a result, S2 has a lower mutational frequency than S1. We recently described monomeric and dimeric SARS-CoV-2 fusion-inhibitory lipopeptides that block viral infection by interfering with S2 conformational rearrangements during viral entry. Importantly, a dimeric lipopeptide was shown to block SARS-CoV-2 transmission between ferrets *in vivo.* Because the S2 subunit is relatively conserved in newly emerging SARS-CoV-2 variants of concern (VOCs), we hypothesize that fusion-inhibitory lipopeptides are cross-protective against infection with VOCs. Here, we directly compared the *in vitro* efficacies of two fusion-inhibitory lipopeptides against VOC, in comparison with a set of seven postvaccination sera (two doses) and a commercial monoclonal antibody preparation. For the beta, delta, and omicron VOCs, it has been reported that convalescent and postvaccination sera are less potent in virus neutralization assays. Both fusion-inhibitory lipopeptides were equally effective against all five VOCs compared to ancestral virus, whereas postvaccination sera and therapeutic monoclonal antibody lost potency to newer VOCs, in particular to omicron BA.1 and BA.2. The neutralizing activity of the lipopeptides is consistent, and they can be expected to neutralize future VOCs based on their mechanism of action.

## OBSERVATION

Infection by SARS-CoV-2, as well as other coronaviruses, requires fusion between the viral envelope and the cell membrane, a process mediated by the viral transmembrane spike (S) glycoprotein. Coronaviruses employ a type I fusion mechanism to gain access to the cytoplasm of host cells (1). SARS-CoV-2 S is a homotrimer (2, 3) and each monomer consists of two subunits: S1, which harbors the receptor-binding domain (RBD) and mediates cell surface attachment, and S2, which contains a cell fusion domain and executes membrane merging. Fusion of the viral envelope with the cell membrane during entry requires conformational rearrangement of the fusion domain within S2. Rearrangement of S2 starts with insertion of the terminus into the target cell and culminates with association of two heptad-repeat (HR) domains of the fusion subunit, one near the amino N terminus (HRN) and the other near the C terminus (HRC), into a compact six-helix bundle (6HB) assembly. Achieving the stable 6HB assembly is required for fusion of the cell membrane and viral envelope (reviewed in references 4 and 5), and the assembly can be inhibited by the use of lipopeptides that interfere with its formation and with subsequent membrane fusion and viral entry (6–8).

We have shown previously that monomeric and dimeric anti-SARS-CoV-2 fusion-inhibitory lipopeptides inhibit viral infection by interfering with S2 structural rearrangements during viral entry (6). A dimeric lipopeptide was shown to block SARS-CoV-2 transmission between ferrets *in vivo* (7) and to be effective *in vitro* against several of the variants of concern (VOCs) that had emerged at that time (February 2021): the alpha and beta variants. Since that time, the emergence of new variants has continued and mutations in S1 have increased resistance to postvaccination sera as well as multiple monoclonal antibody preparations, presenting a challenge to vaccination and treatment strategies (9–14). We conducted a head-to-head comparison of monomeric and dimeric fusion-inhibitory lipopeptides, a panel of seven vaccine immune sera obtained 28 days after second vaccination with BNT162b2, and a commercially available COVID-19 monoclonal antibody against SARS-CoV-2 and VOCs: alpha, beta, delta, and omicron BA.1 and BA.2. We used inhibitory lipopeptides derived from the HRC domain of SARS-CoV-2 S and a lipopeptide derived from the human parainfluenza virus type 3 (HPIV3) F protein HRC domain as a negative control, both described previously (7).

**FIG 1:**
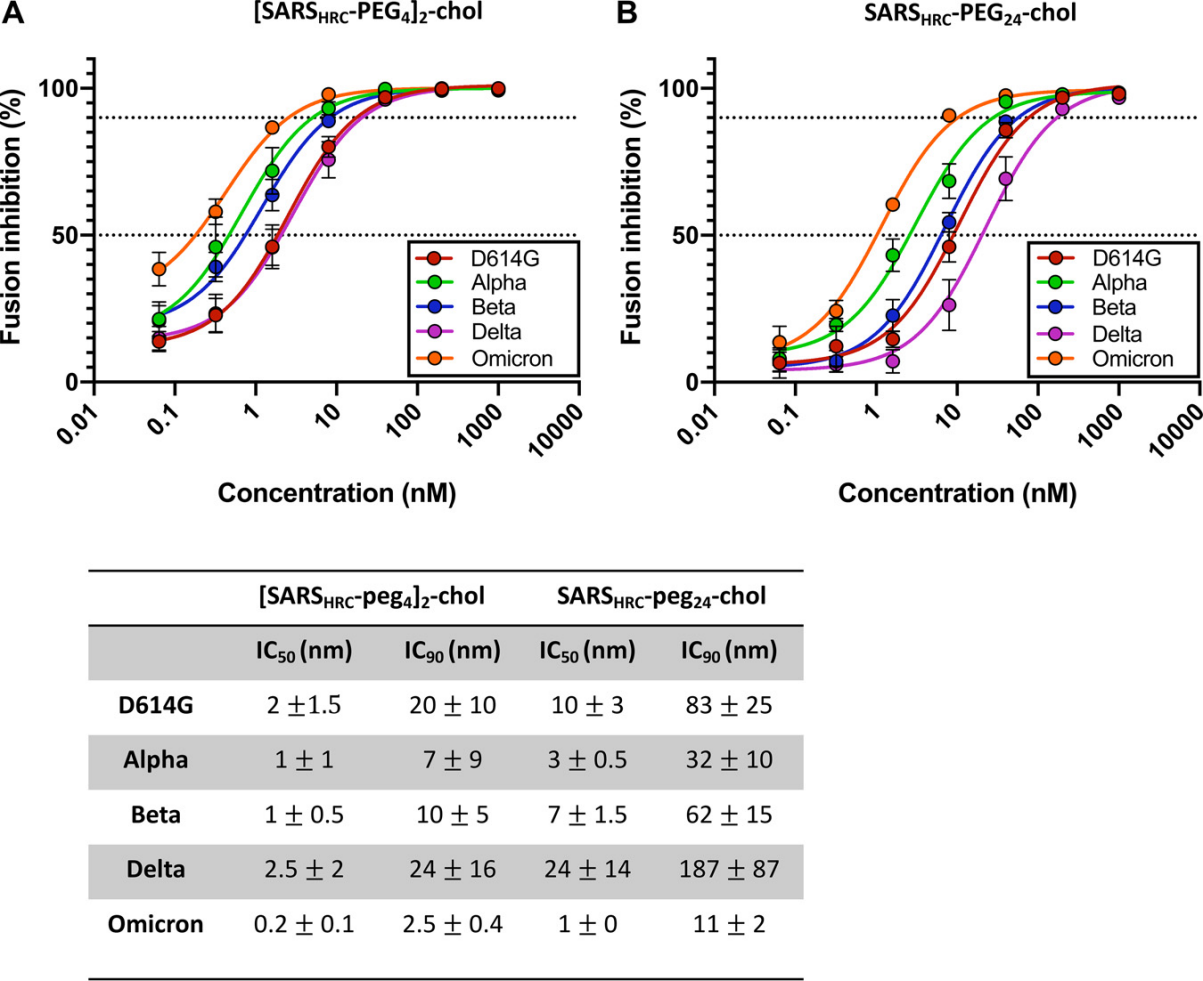
Fusion-inhibitory activity of [SARS_HRC_-PEG_4_]_2_-chol (A) and SARS_HRC_-PEG_24_-chol (B) peptide against emerging SARS-CoV-2 S variants. Inhibitory activity was assessed in an assay based on alpha complementation of β-galactosidase (β-Gal) where hACE2 receptor-bearing cells expressing the omega peptide of β-Gal are mixed with cells coexpressing glycoprotein S and the alpha peptide of β-Gal, and cell fusion leads to alpha-omega complementation (24). Fusion is stopped by lysing the cells, and after addition of the substrate (Tropix Galacto-Star chemiluminescent reporter assay system; Applied Biosystems), luminescence is quantified on a Tecan M1000PRO microplate reader. Fusion between cells expressing SARS-CoV-2 glycoprotein (D614G, alpha, beta, delta, or omicron) and the α subunit of β-galactosidase and human kidney epithelial 293T cells expressing hACE2 receptor and the ω subunit of β-galactosidase was assessed in the presence of different dilutions of inhibitory peptide. The resulting luminescence was quantified using a Tecan Infinite M1000PRO reader. Percent inhibition was calculated as the ratio of relative luminescence units in the presence of a specific concentration of inhibitor and the relative luminescence units in the absence of inhibitor and corrected for background luminescence. % inhibition = 100 × [1 − (luminescence at X − background)/(luminescence in the absence of inhibitor − background)]. Values are presented as mean (±standard error of the mean) from three independent experiments and are shown in the table below.

To determine the impact of S mutations on peptide efficacy, we first examined the ability of lipopeptides to inhibit fusion mediated by each of these emerging S protein mutants in a β-galactosidase (β-Gal) complementation cellular fusion assay (Fig. 1). Briefly, 293T cells expressing human angiotensin-converting enzyme 2 (hACE2) and the N-terminal portion of β-Gal were mixed with cells expressing the various SARS-CoV-2 S proteins (alpha, beta, delta, and omicron BA.1) and the C-terminal portion of β-Gal. When fusion mediated by S occurs, the two portions of β-Gal combine to generate a catalytically active species, and fusion is detected via the luminescence that results from substrate processing by β-Gal. cDNAs coding for human angiotensin-converting enzyme 2 (hACE2) fused to the fluorescent protein Venus, the SARS-CoV-2 S, and all VOC S proteins were cloned in a modified version of pCAGGS containing a puromycin resistance cassette for positive selection. All cDNAs were codon optimized for mammalian expression. In Fig. 1, percent inhibition of cell-cell fusion mediated by each S protein corresponds to the extent of suppression of the luminescence signal that was observed in the absence of any inhibitor (i.e., 0% inhibition corresponds to maximum luminescence signal). The dimeric (A) and monomeric (B) SARS-CoV-2 HRC lipopeptides potently inhibited S-mediated fusion mediated by all the VOCs, with a 50% inhibitory concentration (IC_50_) range from 1 to 24 nM and an IC_90_ range from 11 to 187 nM for the monomer and an IC_50_ range from 0.2 to 2.5 nM and an IC_90_ of ~20 nM for the dimer (IC values shown in the table in Fig. 1). Omicron BA.1 S was the most sensitive to inhibition, inhibited by the dimeric peptide with an IC_50_ of ~0.2 nM. The HPIV3 lipopeptide, used as a negative control, did not inhibit fusion mediated by any S protein at any concentration tested. Thus, SARS-CoV-2 HRC lipopeptides are potent inhibitors of fusion mediated by SARS-CoV-2 S derived from currently identified VOCs.

**FIG 2:**
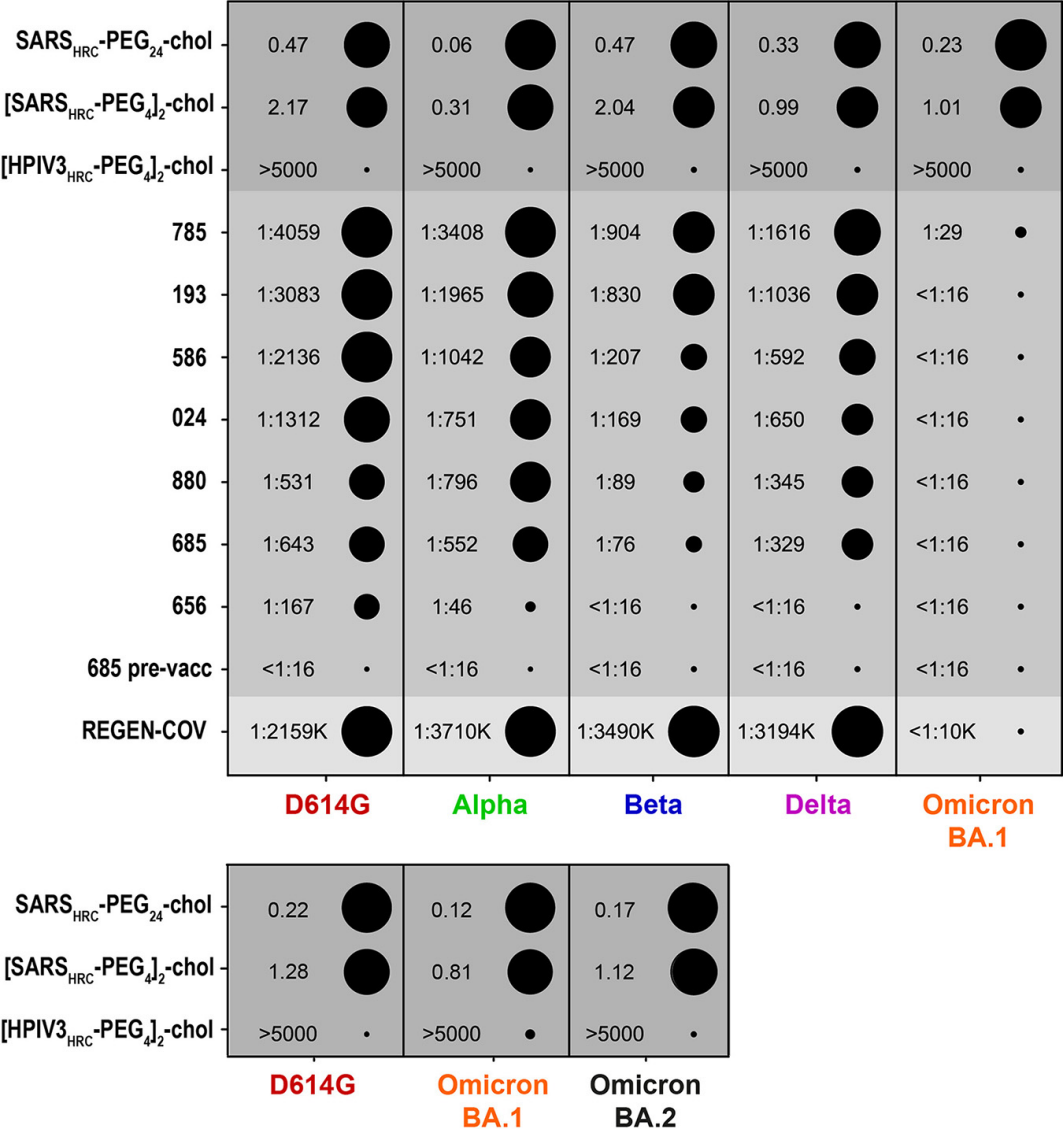

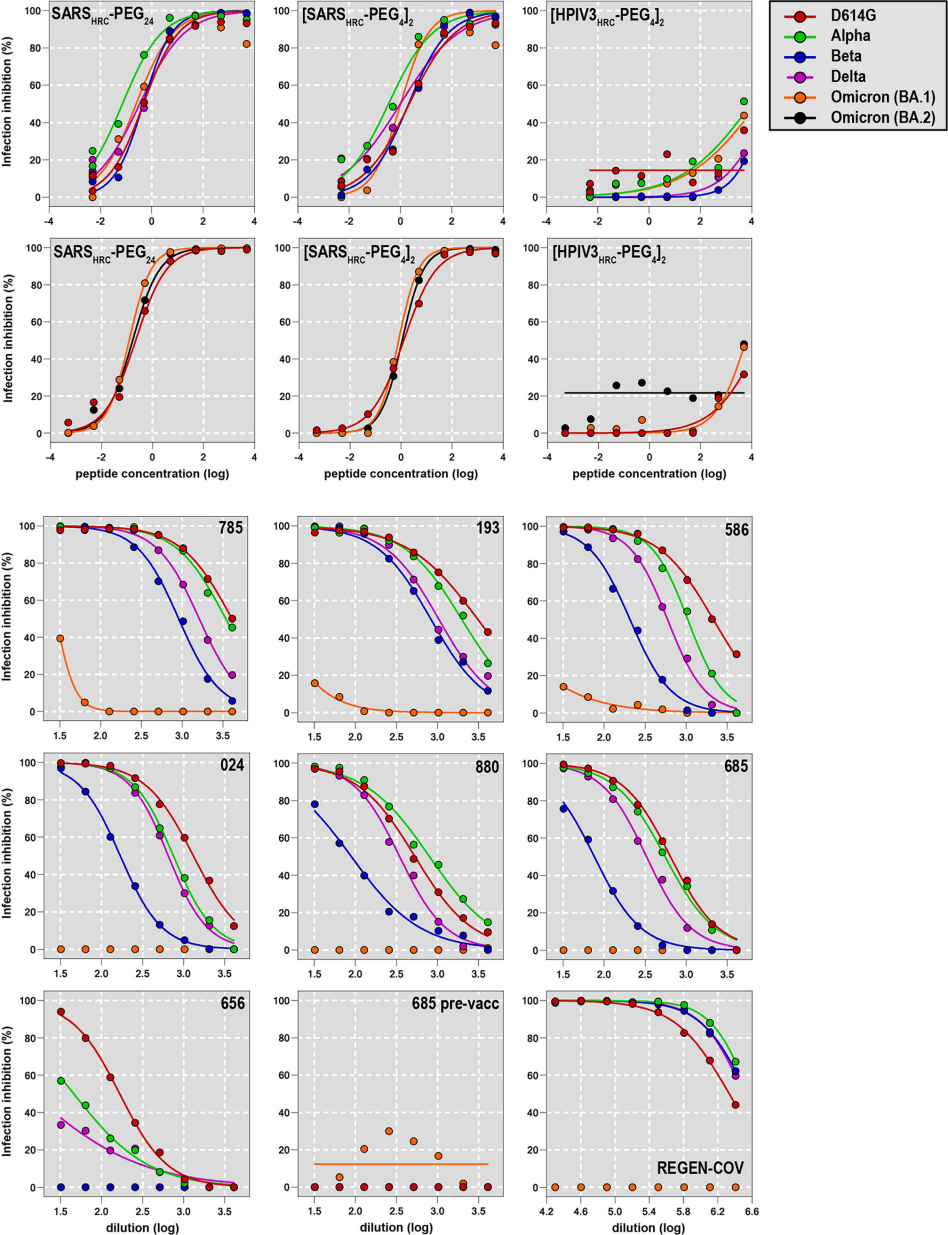
Potency of inhibitory lipopeptides and postvaccination sera against entry of wild-type SARS-CoV-2 and VOCs (D614G, alpha, beta, delta, omicron BA.1, and omicron BA.2). In an 8-h infectious virus entry assay, the efficacy of [SARS_HRC_-PEG_4_]_2_-chol, SARS_HRC_-PEG_24_-chol, and control [HPIV3_HRC_-PEG_4_]_2_-chol peptides (top), seven postvaccination sera, a control prevaccination serum (numbered, bottom), and one commercial SARS-CoV-2 therapeutic monoclonal antibody (REGEN-COV) against ancestral SARS-CoV-2 and VOCs (alpha, beta, delta, and omicron) was determined as previously described (7, 15). Samples were run in triplicate and repeated in three independent experiments. Virus entry was assessed in Calu-3 cells grown in Opti-MEM (Gibco), supplemented with penicillin (100 IU/mL) and streptomycin (100 IU/mL) at 37°C in a humidified CO_2_ incubator. Heat-inactivated sera were 2-fold diluted in Opti-MEM starting at a dilution of 1:16 in 50 μL. Fusion-inhibitory peptides were diluted 10-fold in Opti-MEM starting at a concentration of 5,000 nM. 400 PFU of SARS-CoV-2 (D614G, alpha, beta, delta, omicron BA.1, and omicron BA.2) were added to each well in 50 μL and incubated at 37°C for 1 h. The virus-inhibitor mixtures were then transferred onto the human airway cell line Calu-3 and incubated for 8 h. After incubation, cells were fixed and plaques were stained with polyclonal rabbit anti-SARS-CoV-2 nucleocapsid antibody (Sino Biological) and a secondary peroxidase-labeled goat anti-rabbit IgG (Dako). Signal was developed by using a precipitate-forming 3,3′,5,5′-tetramethylbenzidine substrate (TrueBlue; Kirkegaard & Perry Laboratories), and the number of infected cells was counted per well by using an Immunospot image analyzer (CTL Europe GmbH). Infection controls were included on each plate, and we used one nonreactive fusion-inhibitory peptide (HPIV3) and one prevaccination serum as a negative control in each assay. IC_50_ values were determined by performing four-parameter nonlinear regression with variable slope on normalized and transformed data (GraphPad Prism 9), and potencies were defined within each class based on transformed IC_50_. The response range was log transformed, and strongest to weakest responses were calculated per sample type (inhibitory peptide or sera). The response range was subdivided into 10 ranks with equivalent distances, and each sample was assigned one of these ranks. Ranks were then visualized in SigmaPlot. When no neutralization was observed, we set the value as 1 dilution step lower than the lowest dilution. IC_50_ values for inhibitory peptides are shown in nanomolar concentrations, and IC_50_ values for postvaccination sera are shown as dilutions.

We next assessed the potency of these monomeric and dimeric fusion-inhibitory lipopeptides at inhibiting viral entry of SARS-CoV-2 variants in comparison with a panel of postvaccination sera obtained 28 days after a second shot of BNT162b2, and a commercially available COVID-19 monoclonal antibody, in a live virus entry assay (Fig. 2). Sera were obtained as part of a clinical study in health care workers (15), approved by the institutional review board of the Erasmus MC (medical ethical committee, MEC-2020-0264), which adhered to the principles of the Declaration of Helsinki and in which written informed consent was obtained from every participant. Serum was collected in 10-mL tubes without anticoagulant, centrifuged at 2,500 rpm for 15 min, aliquoted, and stored at −20°C for further experiments. Before use, sera were thawed and heat inactivated at 56°C for 30 min. The SARS-CoV-2 VOCs were grown in Calu-3 cells and sequence confirmed (D614G SARS-CoV-2 [BavPat1/2020 EVAg Ref-SKU: 026V-03883], alpha [MW947280], beta [OM286905], delta [OM287123], omicron BA.1 [OM287553], and BA.2 variants [submitted to Genbank]).

Both lipopeptides showed effective inhibition of fusion for all VOCs tested, with similar efficacy as detected against the ancestral SARS-CoV-2 (D614G). IC_50_ values for the dimeric lipopeptide ([SARS_HRC_-PEG_4_]_2_-chol) were between 0.31 and 2.17 nM (0.31 nM for alpha, 2.17 nM for D614G). The monomer (SARS-CoV-2_HRC_-PEG_24_-chol) was also potent against all VOCs with IC_50_ values below 0.5 nM. Polyclonal postvaccination sera were included as controls and showed a broad reactivity against all SARS-CoV-2 VOCs. However, cross-reactivity of postvaccination sera from individuals who received two doses of BNT162b2 vaccine was markedly reduced against recently emerged VOCs, as expected. Compared to ancestral SARS-CoV-2, we measured overall lower titers to the beta VOC, and neutralization was undetectable in six out of seven postvaccination sera tested against omicron BA.1 at the serum dilutions tested. Of note, while these experiments were conducted using sera from individuals who received two doses of vaccine, it was recently determined that three doses of the BNT162b2 vaccine induces substantial neutralizing activity against omicron BA.1 and BA.2, but even three doses provide 3-fold-less neutralizing activity against omicron than against the Wuhan reference strain (16). This study also examined only BNT162b2 vaccine sera; other vaccine sera may differ in activity. The commercial COVID-19 monoclonal antibody (REGN) control was effective in this assay against the alpha, beta, and delta variants but failed to neutralize omicron BA.1 and BA.2, as shown previously (13). Neutralization titers against the alpha and delta variants were similar to those against the ancestral SARS-CoV-2 strain.

The SARS-CoV-2-specific antiviral lipopeptides (monomeric or dimeric) showed equal efficacies against ancestral SARS-CoV-2 and the alpha, beta, delta, and omicron BA.1 and BA.2 variants. This head-to-head comparison indicates that these fusion inhibitors provide consistent entry blockade even of variants that escape antibody inhibition and are not subject to the constant evolution of the S1 domain that is a hallmark of SARS-CoV-2 evolution.

Our published sequencing analysis revealed no significant mutations in viruses emerging from SARS-CoV-2 HRC lipopeptide-treated airway tissues, suggesting that no resistance mutations emerged under the selective pressure of lipopeptide treatment after 7 days of treatment (6). In future studies, we will use infections in the human airway epithelial (HAE) model as well as animal models to study the potential for emergence of SARS-CoV-2 viruses resistant to the inhibitory effect of our peptides after longer periods of treatment or repeated courses, as we have done for other viruses (17, 18).

The efficacy of SARS-CoV-2-inhibitory peptides at blocking viral transmission (7, 19, 20), taken together with our published data for other viruses (21–26), suggests that an effective antiviral effect can be achieved via administration of antiviral peptides. The potency of these lipopeptides for neutralization of SARS-CoV-2 VOCs is substantial and consistently observed across the VOCs. The value of this approach is that the neutralizing activity of the lipopeptides is consistent, and therefore, they may be expected to neutralize future VOCs based upon their mechanism of action. This feature highlights the advantage of fusion-inhibitory peptides and their potential as a reliable intervention strategy aimed at inhibition of virus transmission.
